# *Streptomyces* Pigmented Extract-Loaded Nanoemulsions: Stability, Antibacterial Activity, and Cytotoxicity Assessment in Skin Cells

**DOI:** 10.3390/molecules31142537

**Published:** 2026-07-21

**Authors:** Juanita Rojas Cortés, Luis Eduardo Díaz Barrera, María Ximena Quintanilla-Carvajal

**Affiliations:** 1 Processes Design & Management, Faculty of Engineering, Universidad de La Sabana, Campus Universitario del Puente del Común, Km7 Autopista Norte de Bogotá, Chía 250047, Cundinamarca, Colombia; juanitaroco@unisabana.edu.co; 2 Grupo de Investigación en Procesos Agroindustriales, Faculty of Engineering, Universidad de La Sabana, Campus Universitario del Puente del Común, Km7 Autopista Norte de Bogotá, Chía 250047, Cundinamarca, Colombia; luis.diaz1@unisabana.edu.co

**Keywords:** nanoemulsion, methicillin-resistant, *Staphylococcus aureus*, *Streptomyces*, microfluidization, pigmented extract, stability

## Abstract

*Streptomyces*-derived pigments are promising bioactive metabolites for cosmeceutical applications due to their antimicrobial potential; however, their stability may be affected by environmental conditions. This study evaluated the antibacterial activity, physicochemical characterization, pH stability, cytotoxicity, and nanoemulsion-based protection of a pigmented extract from *Streptomyces*. Pigment production was maintained during culture scale-up, with the highest antibacterial response observed between the third and fourth weeks, mainly in the supernatant fraction. The extracted pigment showed concentration-dependent activity against methicillin-resistant *Staphylococcus aureus* (MRSA), with a minimum inhibitory concentration (MIC) of 0.05% and an IC_50_ of 0.293%. FTIR analysis suggested the presence of phenolic hydroxyl groups, aromatic structures, and conjugated systems, while pH analysis indicated greater stability between pH 5 and 8. A Box–Behnken response surface design was used to develop macroemulsions and nanoemulsions by microfluidization, evaluating microfluidization pressure, oil proportion, and pigmented extract concentration. Nanoemulsions showed droplet sizes from 243.4 to 902.4 nm, polydispersity indices from 0.326 to 0.471, and zeta potential values below −30 mV. After two months of refrigerated storage, average droplet size, polydispersity, and zeta potential remained stable, although antibacterial activity decreased slightly over time. The most stable pigment-loaded nanoemulsions were non-cytotoxic to HaCaT and HDFa skin cells under in vitro conditions, maintaining cell viability above 78%. These findings support microfluidized nanoemulsions as protective delivery systems for *Streptomyces*-derived pigments with potential cosmeceutical applications.

## 1. Introduction

*Streptomyces* is a prolific source of bioactive secondary metabolites and pigmented extracts that serve as natural ingredients for food, textiles, cosmetics, and pharmaceutical applications. These metabolites exhibit diverse activities, including antimicrobial, antioxidant, cytotoxic, antifungal, antiviral, antitumoral, antihypertensive, and, most prominently, antibiotic and immunosuppressive effects, supporting their relevance for skin-care formulations and, notably, anti-MRSA activity [1,2]. Methicillin-*resistant Staphylococcus aureus* (MRSA) is a pathogenic bacterium that colonizes healthy skin and mucosal surfaces and can cause a broad spectrum of skin and soft-tissue infections [2,3,4]. It is also implicated in inflammatory dermatoses, including acne vulgaris, rosacea, and hidradenitis suppurativa, conditions frequently managed with systemic antibiotics such as doxycycline or minocycline for their rapid anti-inflammatory and antibacterial effects; however, repeated antibiotic use accelerates resistance. MRSA commonly exploits microtrauma, such as wounds, where surface adhesins bind extracellular matrix proteins, promoting bacterial proliferation and increasing susceptibility in these patient groups [5]. Beyond healthcare settings, community transmission is widespread, and the pathogen’s capacity to adapt and generate increasingly resistant clones presents an ongoing challenge [4].

However, these properties are prone to degradation or inactivation, which motivates the encapsulation of pigmented extracts to extend shelf life. Nanoencapsulation involves entrapping nano-sized solids, liquids, or gases (the core) within a surrounding matrix or wall material, typically with characteristic dimensions around 200 nm; the goal is to protect bioactive compounds in the core and enable controlled release by diffusion or by triggers such as shear, pH shifts, or enzymatic action [6]. Emulsion systems provide a widely used encapsulation platform. Nanoemulsions are colloidal dispersions of water in oil or oil in water with droplet diameters on the order of 100 nm [7,8,9]. Several studies have shown that the type of emulsifier can influence emulsion stability. In this regard, soy lecithin is widely used in the food and pharmaceutical industries because it is edible and has low allergenic potential [10]. Nanoemulsions can enhance the bioavailability of many actives and offer favorable attributes, including kinetic stability, rapid digestibility, reduced degradation, and controlled release [7]. In cosmetics, nanoemulsions are broadly applied in anti-aging, moisturizing, and skin-brightening products because they promote skin penetration, disperse readily, support controlled release, which allows for diverse formulations, and maintain long-term stability [11].

Several high-energy methods are used to produce nanoemulsions, including high-pressure homogenization (HPH), ultrasonication, and microfluidization [12]. Microfluidization can yield nanoemulsions with high temporal stability, support continuous processing, and frequently achieve target sizes in a single pass [13]. In this technique, a positive-displacement pump forces the pre-emulsion through micro-channels into an interaction chamber, where intense shear, cavitation, and impingement break droplets into the nanometric range [12]. The process parameters, particularly the pressure and number of passes (cycles), must be optimized to achieve the desired droplet size, polydispersity, encapsulation efficiency, and release profile [14]. Droplet size and its distribution are primary determinants of kinetic stability: at a given coalescence rate, smaller droplets separate more slowly, thus conferring greater stability [15]. Formulation variables are also important; the composition of the continuous phase, the concentration of solutes in the dispersed phase and the emulsifier system can alter viscosity and interfacial coverage, thereby modulating stability [14]. Storage conditions should be controlled to prevent phase separation and preserve the bioactivity of the pigmented extract, as light exposure and temperature can induce physicochemical changes in nanoemulsions [16].

Given the importance of encapsulating active compounds, such as pigmented *Streptomyces* extracts with documented antibacterial activity against methicillin-resistant *Staphylococcus aureus* (MRSA), this study aimed to evaluate emulsion stability and anti-MRSA activity of lecithin-stabilized pigment-loaded nanoemulsions. The factors examined included the proportion of the continuous phase, extract concentration, and microfluidization conditions. Stability was measured at time zero and after two months of storage and was monitored over 2 weeks under two temperature regimens.

## 2. Results and Discussion

### 2.1. Bacterial Strain and Culture Conditions

The selected *Streptomyces* strain showed visible growth under the evaluated culture conditions, accompanied by the production of a yellow-pigmented metabolite in ISP2 medium. Pigment production was initially observed after incubation in small-volume cultures and was maintained during the sequential scale-up. The persistence of pigmentation throughout the scaling stages suggested that the strain retained its metabolic capacity under increased culture volumes. In addition, antibacterial activity assays performed at each scaling step allowed for the verification of the inhibitory potential of the produced metabolites prior to extract recovery.

**Figure 1:**
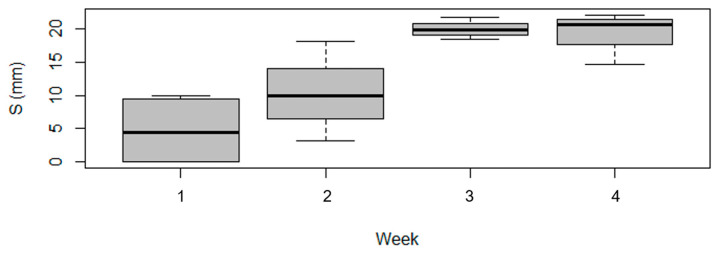
Box plot of MRSA inhibition halo diameters produced by the supernatant fraction over the incubation period.

The antibacterial activity of the culture fractions was evaluated against methicillin-resistant *Staphylococcus aureus* (MRSA) using the disc diffusion method. A 33.3% ratio of MRSA inoculum to culture supernatant was maintained during the assay. As shown in Figure 1, inhibition halo diameters increased progressively during the first weeks of incubation, suggesting an increased accumulation or secretion of antibacterial secondary metabolites into the culture medium over time. This behavior may be associated with the activation of microbial metabolic responses under environmental or nutritional stress conditions, which can promote the biosynthesis of bioactive secondary metabolites in *Streptomyces* species [17].

The supernatant showed a marked increase in antibacterial activity from week 1 to week 3, reaching its highest average inhibition diameter in week 3, with a value of 20.07 ± 1.71 mm. A similar response was observed for the biomass fraction, although the maximum inhibition diameter was lower than that observed for the supernatant. These results indicate that both biomass-associated and extracellular metabolites contributed to MRSA inhibition; however, the supernatant exhibited the strongest inhibitory effect. This finding supports the use of the supernatant as the preferred fraction for metabolite extraction, since many bacterial secondary metabolites are secreted into the extracellular medium during microbial growth, facilitating their recovery and potentially increasing their concentration in the extract [18]. Moreover, the inhibition values obtained in this study are consistent with previous reports, who described inhibition halos of approximately 19.33 ± 0.58 mm for *Streptomyces* extracts [17]. In the present study, the maximum inhibition response was observed in week 3 using the supernatant fraction, highlighting the antibacterial potential of the selected *Streptomyces* strain against MRSA.

To statistically evaluate the effect of incubation time on the inhibition halos produced by the supernatant, each week was considered as an experimental factor. Prior to ANOVA, normality was assessed using the Shapiro–Wilk test, which is recommended for small sample sizes [19], and homogeneity of variance was evaluated using the Breusch–Pagan test. The obtained *p*-values, 0.9175 for normality and 0.3805 for homoscedasticity, indicated no significant violation of the assumptions required for one-way ANOVA. The ANOVA showed a statistically significant effect of incubation time on MRSA inhibition halo diameter (*p* = 0.0101). Tukey’s HSD post hoc test revealed significant differences between week 1 and week 3 (*p* = 0.0161), as well as between week 1 and week 4 (*p* = 0.0220), indicating that antibacterial activity increased significantly after the initial incubation period.

As shown in Figure 1, the box-and-whisker plot illustrates the time-dependent increase in inhibition halo diameter. The mean inhibition halo in the initial week was markedly lower than those observed in weeks 3 and 4, while the variability decreased in later weeks, suggesting a more consistent antibacterial response after sufficient incubation time. Overall, these findings indicate that the production and/or accumulation of antibacterial metabolites by *Streptomyces* was time-dependent, with the strongest inhibitory effect observed in the supernatant fraction around week 3.

### 2.2. Pigment Characterization

#### 2.2.1. pH Titration

**Figure 2:**
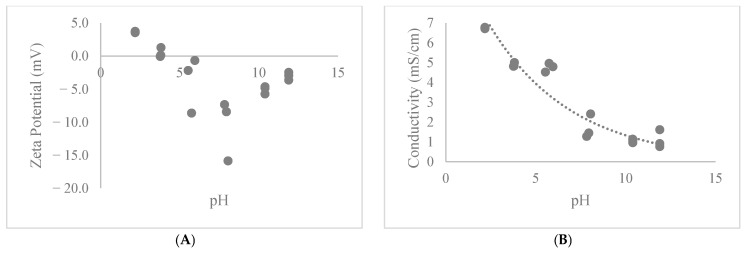
Effect of pH on the zeta potential (**A**) and conductivity (**B**) of the pigment extracted from *Streptomyces*.

A pH titration was conducted to assess the stability of the compound prior to encapsulation. As shown in Figure 2, the zeta potential (ζ) decreases as the pH increases, reaching a minimum at pH 8, after which it begins to rise again. This trend aligns with literature, where it is reported that pigments generally exhibit negative zeta potentials [20,21]. It is desirable for the zeta potential to be far from 0, with values lower than −30 mV indicating good stability. The high absolute value of zeta potential reflects stronger electrostatic repulsion between particles, preventing aggregation and thus maintaining system stability [22].

Additionally, electrical conductivity measurements were performed to evaluate the compound prior to emulsification. As illustrated in Figure 2, conductivity decreases with increasing pH, stabilizing around pH 8. It has been established that water-in-oil (W/O) emulsions require low conductivity for stability, as low conductivity suggests efficient encapsulation of the hydrophilic material, in this case, the yellow pigment extract [23]. This is because aqueous phases are generally more conductive than oily solvents [24].

Based on the data from both zeta potential and conductivity graphs, the optimal pH range for encapsulation was found to be between 5 and 8. This range is advantageous, as it is close to the natural pH of facial skin, which is approximately 5.5 [25].

#### 2.2.2. Fourier-Transform Infrared Spectroscopy (FTIR-ATF)

**Figure 3:**
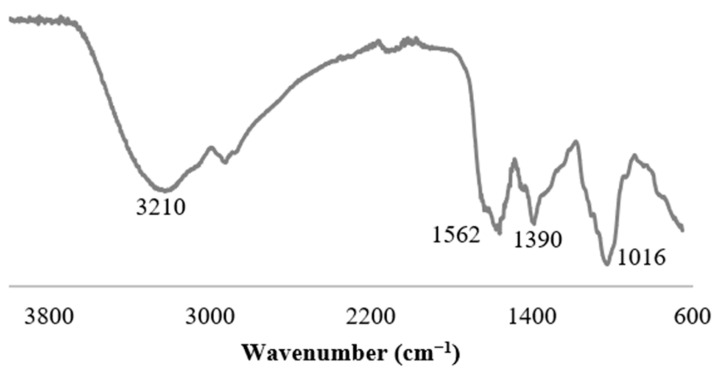
FTIR spectrum of the water-extracted pigment from *Streptomyces*.

The FTIR spectrum was analyzed to identify the main functional groups present in the pigment extracted from *Streptomyces*, based on the interaction of infrared radiation with molecular bonds, which induces changes in dipole moments through vibrational and rotational transitions [26]. As shown in Figure 3, the spectrum exhibited distinct absorption bands associated with hydroxyl, aliphatic, and aromatic structures. A broad absorption band around 3210 cm^−1^ was attributed to O–H stretching vibrations, commonly associated with hydroxyl groups, particularly phenolic structures. This band is consistent with the typical absorption region reported for phenolic O–H groups between 3400 and 3200 cm^−1^. Similar FTIR profiles have been reported for pigment-rich fractions obtained from *Streptomyces,* in which broad O–H stretching bands in the 3400–3200 cm^−1^ region were associated with phenolic groups, supporting the presence of hydroxylated aromatic compounds in these extracts [27].

Additional absorption bands observed between 1600 and 1500 cm^−1^ were assigned to C=C stretching vibrations, suggesting the presence of aromatic rings or conjugated double-bond systems. Meanwhile, bands in the 1460–1375 cm^−1^ range were associated with C–H bending vibrations, which may indicate aliphatic chains or side groups in the pigment structure [28]. These findings are also consistent with previous reports describing aromatic C=C stretching bands between 1600 and 1500 cm^−1^ and C–H stretching bands around 2925–2850 cm^−1^ in *Streptomyces*-derived pigment-rich fractions, suggesting the coexistence of aromatic systems and aliphatic side chains [27].

Overall, the spectral features observed in the extracted pigment suggest the presence of aromatic rings, phenolic hydroxyl groups, and conjugated chemical structures. These functional groups are relevant because phenolic and aromatic compounds have been widely associated with antimicrobial activity. In this regard, the antibacterial activity observed against MRSA may be partially related to the presence of these bioactive chemical groups. Furthermore, previous studies on pigment-rich fractions from *Streptomyces* have reported UV–Vis, FTIR, and LC–MS evidence supporting the presence of yellow–orange 1,4-naphthoquinone-like polyketides, including compounds such as juglomycin Z, WS-5995B, and naphthopyranomycin. These metabolites have been associated with broad biological activity, including antibacterial, antioxidant, anti-inflammatory, and depigmenting effects [27].

The presence of phenolic and conjugated aromatic structures in the FTIR spectrum therefore supports the hypothesis that the extracted pigment contains bioactive secondary metabolites with antimicrobial potential. This interpretation agrees with previous reports indicating that phenolic structures and *Streptomyces*-derived polyketides may contribute to antibacterial effects [17]. In particular, 1,4-naphthoquinone-like polyketides have been described as compounds capable of inhibiting Gram-positive and Gram-negative bacteria, potentially through disruption of bacterial cell walls or inhibition of essential bacterial enzymes. Therefore, the functional groups identified in the present FTIR analysis are compatible with chemical motifs previously associated with the antimicrobial properties of *Streptomyces* pigments [27].

#### 2.2.3. Minimum Inhibitory Concentration of the Extracted Pigment

**Figure 4:**
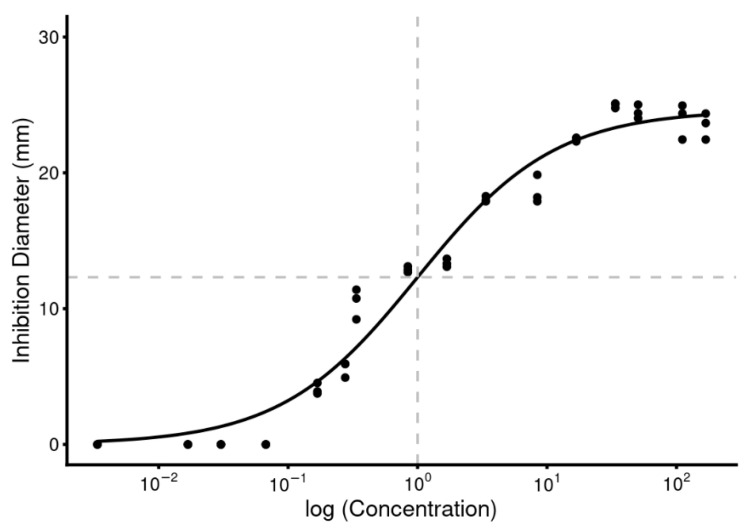
Concentration-dependent inhibition of MRSA by the pigment extracted from *Streptomyces*. The dashed line indicates the IC_50_ of the extracted pigment.

The antibacterial activity of the pigment extracted from *Streptomyces* was evaluated at different concentrations against methicillin-resistant *Staphylococcus aureus* (MRSA). As shown in Figure 4, the inhibition response followed a concentration-dependent pattern, in which low pigment concentrations produced no detectable or minimal inhibition halos, whereas increasing concentrations resulted in a progressive enlargement of the inhibition diameter.

At the lowest tested concentrations, no measurable inhibition was observed, suggesting that the pigment concentration was insufficient to affect MRSA growth under the evaluated assay conditions. However, inhibition became evident from 0.05%, which was identified as the minimum inhibitory concentration (MIC) of the extracted pigment. The MIC is defined as the lowest concentration of an antimicrobial agent capable of inhibiting the in vitro growth of a given bacterial strain [29]. From this concentration onward, the inhibition halo diameter increased progressively, indicating that the antibacterial effect of the pigment was concentration dependent.

The response curve showed a sigmoidal-like behavior, characterized by an initial low-response region, followed by a rapid increase in inhibition diameter at intermediate concentrations and a tendency toward stabilization at higher concentrations. The IC_50_ value, defined as the concentration required to reach 50% of the maximum inhibitory response [30], was estimated at 0.293%. This value indicates that relatively low concentrations of the pigment were sufficient to produce a substantial inhibitory effect against MRSA.

At higher concentrations, the inhibition halos reached approximately 23–25 mm, suggesting that the antibacterial response approached a maximum effect. This plateau may be associated with the maximum effective concentration under the experimental conditions. Overall, the MIC and IC_50_ values confirmed that the extracted pigment exhibited concentration-dependent antibacterial activity, supporting its potential use as a bioactive antimicrobial compound.

### 2.3. Emulsion Preparation

The significant factors influencing macroemulsions (MEs) and nanoemulsions during the first (C1) and second (C2) cycles were evaluated to identify the conditions that promoted greater stability. In MEs, pigment concentration and its interaction with oil proportion significantly influenced the polydispersity index (PDI) and average droplet size (ADS). Both main factors were significant for ζ-potential as shown in Table 1. This behavior may be explained by partial crystallization in the dispersed phase, in which droplets undergo partial coalescence [31]. Under these conditions, the morphology and physical state of the pigment can modify interfacial structure and compromise emulsion stability. As pigment concentration increased, ADS and PDI values increased, while the ζ-potential approached 0 mV, indicating reduced electrostatic stabilization and overall stability. Significant differences were observed between the 7% pigment group and both the 0% and 3.5% groups. For PDI, *p*-values were 0.0054 (7% vs. 0%) and 0.0179 (7% vs. 3.5%), while for ADS, they were 0.000855 (7% vs. 0%) and 0.0023 (7% vs. 3.5%). The marked increase at 7% supports enhanced droplet coalescence. These findings indicate that pigment-free emulsions exhibit superior stability within the studied formulation range.

Oil proportion is also relevant for two main reasons: (i) changing the oil fraction alters the effective (relative) pigment concentration; and (ii) increasing the oil fraction raises the continuous-phase viscosity, which lowers creaming or sedimentation tendencies [32]. Consistent with the above, oil proportion showed clear associations with all responses. For the ζ-potential, higher oil proportions were more favorable, with a significant difference between 90–80% (*p*-value = 0.0122) and 90–85% (*p*-value = 0.0440). For PDI and ADS, emulsions without pigment (0%) performed better at lower oil proportions, whereas at 7% pigment, higher oil fractions performed better. A plausible mechanism is that, although increased continuous-phase viscosity generally suppresses creaming/sedimentation, high solute loading can promote crystallization and partial droplet coalescence, thereby requiring a greater oil fraction to counteract these effects [31]. Pairwise contrasts that reached significance frequently involved the 7–pigment:80–oil condition, which exhibited the highest ADS.

For C1, the models for polydispersity index (PDI) identified microfluidization pressure (C) as a significant main effect. For ADS, the oil fraction (B), microfluidization pressure (C) and the AC interaction were also significant, indicating that the effect of microfluidization pressure depends on the pigment concentration. For the ζ-potential, all A, B and C were significant, with significant AC interaction. After the first microfluidization pass, the system transitioned to a nanoemulsion, and the interfacial and size metrics were predominantly governed by the applied pressure. Increasing microfluidization pressure consistently improved stability. For PDI, the contrast between 0 and 20,000 psi was significant (*p*-value = 0.0066), with the best performance at 20,000 psi, while ADS presented 0–20,000 (*p*-value = 0.0136) and 0–10,000 psi (*p*-value = 0.0296) as significant pairs. For zeta potential, significant differences were detected between 0 and 10,000 psi (*p*-value = 0.0121). The ζ potential model also showed significant pressure and pigment interactions. At higher pressures, formulations with greater pigment loading exhibited larger absolute ζ potential values, that is, values farther from 0 mV. A plausible explanation is that higher pressure generates smaller droplets and greater interfacial area [33], which enhances the adsorption of pigment-associated surface-active components and promotes more effective interfacial charge development. Multiple pairwise contrasts relative to the 7-percent pigment at the 0 psi condition was significant; that condition yielded the lowest ζ potential magnitude. Also, for ζ potential, oil proportion was a significant factor; the same trend was observed with ME, with better performance in 90%.

Regarding the ζ potential, it was found that with pressure, the values moved further away from 0 mV, and regarding the pigment concentration, it was found that a lower pigment concentration had a better response in the variable, which is consistent with the above; however, it did not present a significant difference. The ζ potential is the potential at the ion surface of the thin liquid layer attached to the surface of the particles. This particle charge acts as an electrical barrier that prevents particle coalescence, thus helping to stabilize the suspension; therefore, it can be positive or negative [34].

In nanoemulsion systems, ζ-potential values of ~20–30 mV are considered optimal because they reflect a strong electrostatic repulsion between droplets and good colloidal stability. Emulsions with ζ-potential values near 0 mV tend to be unstable, leading to rapid flocculation or coagulation during storage, whereas values further from 0 mV confer greater stability because the repulsive forces dominate the attractive forces. For PDI, lower values are desirable because they indicate a more uniform droplet-size distribution, favoring colloidal stability [35].

For ADS and PDI, lower oil proportions performed better, in contrast to the macroemulsion results. A likely explanation for this is the properties of the oil used (HOPO), which contains thermosensitive components [36]. During microfluidization, shear-induced heating can elevate the sample temperature [37]; under these conditions, HOPO’s thermosensitive constituents may weaken interfacial stabilization and/or alter viscosity, diminishing droplet break-up efficiency at higher oil contents and leading to larger sizes and broader distributions.

In addition, increased sample viscosity likely contributed to the observed behavior. Higher viscosity requires greater energy input in the microfluidizer and reduces size-reduction efficiency. Because the oil fraction elevates emulsion viscosity, larger oil proportions hinder droplet disruption and yield larger ADS at fixed operating conditions. At lower viscosities, the droplets experience higher effective strain rates in the microfluidization chamber and are more readily disrupted, resulting in smaller sizes under identical settings [37].

For C2, the significant factors of the model (*p* < 0.05) are shown as a function of each of the four response variables. In the case of inhibition diameter (ID), pigment concentration was identified as the only significant factor for all cases described, both in macroemulsion and in the nanoemulsion with one and two cycles. This finding is consistent, as a study showed that pigment concentration directly influences ID [38]. Natural pigments extracted from microorganisms of the genus *Streptomyces* contain secondary metabolites with potent antimicrobial effects against methicillin-resistant *Staphylococcus aureus* (MRSA) [2]. *Streptomyces* produces pigments with metabolites that can effectively inhibit MRSA growth [39]. The ID values increased progressively with increasing pigment concentration. For nanoemulsions, a significant statistical difference was observed between 0% and 7% pigment levels, with *p* = 0.0012 for C1 and *p* = 0.0005 for C2. In contrast, the macroemulsion (ME) exhibited significant differences across all pairwise comparisons: 0% vs. 7% (*p* = 0.0000268), 0% vs. 3.5% (*p* = 0.0000816), and 3.5% vs. 7% (*p* = 0.0252).

The maximum inhibition zone observed for the emulsion formulation was approximately 13.40 mm, which was lower than the 19.33 ± 0.58 mm reported for *Streptomyces* extracts against MRSA [17]. This reduction may be explained by the lower effective amount of freely available pigment in the emulsion system, even when the same nominal sample concentration was evaluated. Since the antibacterial activity of the pigment is concentration-dependent, a lower available fraction of active compounds would be expected to reduce the inhibition zone diameter.

It is also important to consider that inhibition zones obtained using the disk diffusion method should be interpreted with caution, as this assay depends not only on antimicrobial potency but also on the water solubility of the active compounds and their diffusion through the agar medium. Therefore, microorganisms exhibiting larger inhibition zones are not necessarily those with the lowest minimum inhibitory concentration values since the inhibition diameter can be influenced by the solubility, volatility, and diffusion capacity of the tested compound [40]. In this context, the encapsulation of the pigment within the emulsion, particularly given its water-in-oil (W/O) nature, may have reduced the freely diffusible fraction of bioactive compounds and slowed their mass transfer into the agar medium, resulting in a smaller inhibition zone compared with the free extract.

In addition, the antimicrobial behavior of nanoemulsions is strongly influenced by formulation-related factors, including the oil/water/surfactant ratio and the emulsification process, which determine physicochemical properties such as droplet size, polydispersity, interfacial characteristics, and release behavior. These properties can modify the interaction of the formulation with bacterial cells and alter the apparent antimicrobial response [40]. Therefore, the smaller inhibition diameter observed for the emulsion likely reflects lower active compound availability, modified release behavior, and formulation-dependent diffusion limitations under agar diffusion conditions.

In contrast, microfluidization pressure was the most significant factor to describe the variable responses. Increasing pressure reduced ADS and PDI and shifted ζ-potential farther from 0 mV, indicating enhanced electrostatic stabilization. Both contrasts (0 vs. 10,000 psi and 0 vs. 20,000 psi) showed statistically significant differences for ADS (*p* = 0.0432 and *p* = 0.0012, respectively) and PDI (*p* = 0.0193 and *p* = 0.0072, respectively), as well as for ζ-potential (*p* = 0.0151 and *p* = 0.0113, respectively), confirming the strong effect of microfluidization pressure on emulsion stability. These findings agree with a study that reported that electrostatic repulsion, measured by a higher absolute zeta potential, increases with microfluidization pressure [41]. Similarly, a concurrent decrease in droplet size was observed, indicating the same directional association. Consistent with this pattern, higher pressure corresponded to smaller ADS, lower PDI, and larger absolute zeta potential. Within the tested range, 20,000 psi provided the best performance. Furthermore, PDI and ADS presented the interaction between pigment and microfluidization pressure as a significant factor, because higher pigment concentration in emulsions with microfluidization pressure had better performance, as was shown in C1.

Microfluidization pressure plays a key role in determining the ADS, PDI, and ζ-potential, as previous research has shown that increasing the pressure during microfluidization significantly decreases the particle size [33]. This process applies high pressure, which introduces a large amount of energy into the emulsion, which travels through the microchannels at high speeds [42], causing intense shear forces that reduce the particle size and alter the particle size distribution, which, in turn, affects the PDI value [33]. In addition, pressure changes were observed to modify the ζ-potential, possibly due to the surface reorganization of the colloidal system components induced by microfluidization [42].

The results obtained for average droplet size (ADS) and polydispersity index (PDI) are consistent with values reported in the literature for nanoemulsions [42]. Higher microfluidization pressures and the application of a second processing cycle resulted in smaller droplet sizes and narrower size distributions, with the most favorable results observed at 20,000 psi in cycle 2. This confirms that the trends observed in Table 2 are consistent with the expected behavior and with findings reported in previous studies.

As shown above, the number of microfluidization cycles is a key determinant of emulsion stability. Systems subjected to microfluidization exhibited lower ADS, lower PDI, and greater absolute zeta potential. Furthermore, as the number of microfluidization cycles increases, these variables tend to decrease even further. This behavior is consistent with previously reported results, which showed that a greater number of cycles in the microfluidizer significantly decreases droplet size and improves emulsion stability [33]. This is due to the longer exposure time of the pre-emulsions to the high energy provided by the equipment, which further reduces the droplet size of the emulsion. Significant differences in PDI and ADS were observed between the macroemulsion and the two-cycle nanoemulsion. For zeta potential, significant differences were detected across all pairwise comparisons. The predictive equations obtained from the response surface models are summarized in Table 3.

Among the evaluated systems, the optimized C2 formulation was confirmed as the most suitable candidate because it provided the best compromise among reduced droplet size, acceptable PDI, improved zeta potential, physical stability, and antibacterial activity, as presented in Table 3. Model verification further demonstrated good agreement between predicted and experimental values for most of the evaluated responses, with deviations below 10% in most cases.

Finally, the response surface results suggested a slight trend in which smaller droplet sizes were associated with higher inhibition diameters. The optimized C2 formulation presented an average droplet size of 186.2 nm, remaining within the nanoscale range and close to particle sizes previously reported for antibacterial nanocarriers, such as vancomycin-loaded nanoparticles with an effective size of 106.9 ± 1.4 nm [43]. This nanoscale dimension may be favorable for antibacterial performance because smaller droplets can provide a larger surface area, improve dispersion, and enhance the availability of the bioactive compounds at the bacteria–formulation interface.

Although vancomycin inhibits bacterial growth mainly by disrupting cell wall synthesis, the Streptomyces pigment extract may exert antibacterial effects through interactions with the bacterial envelope or related membrane/cell wall structures, as suggested previously [27]. Therefore, the reduced droplet size observed in the optimized C2 formulation may contribute to improved contact between the encapsulated pigment and MRSA cells. In this context, nanoscale properties may support delivery, solubilization, formulation stability, tissue interaction, and antibacterial performance [44].

### 2.4. Physical Stability in Time over Time at Different Temperatures

In this section and subsequent analyses, the emulsions obtained from the experimental runs of the second microfluidization cycle (C2) were evaluated, as this cycle showed the best stability performance in the previous analysis. Under room-temperature storage conditions, the greatest phase separation on day 13 was observed in emulsions containing lower pigment proportions. The purpose of this analysis was to monitor visual changes and phase separation behavior under different storage temperatures over time. Therefore, this section focuses only on physical stability indicators, such as phase separation, creaming, sedimentation, or visible destabilization.

However, the formulations that showed the highest phase separation from the first days were those without microfluidization cycles, indicating a lower intrinsic stability of these emulsions. This behavior is consistent with reported, which observed rapid destabilization in emulsions prepared in a single step with an Ultra-Turrax homogenizer, compared to systems subjected to additional high-energy processes [14]. Consistently, it has also been reported that microfluidization pressure has a decisive influence on stability: emulsions processed at higher pressures show less phase separation and superior physical stability during storage [41]. This is consistent with the trends observed in this study.

Regarding the formulation, emulsions without pigment tended to develop greater and more sustained phase separation over time. This could be attributed to the presence of pigment in the internal aqueous phase, which increased the viscosity of the system and contributed to delaying creaming or coalescence phenomena, as has been proposed for other systems with encapsulated hydrophilic solutes [45]. Overall, at room temperature, lower phase separations were observed in formulations with higher microfluidization pressure and pigment content, consistent with the literature highlighting the combined role of processing intensity and composition in the stabilization of multiple emulsions.

In terms of temporal evolution, significant differences were found in phase separation between day 4 and days 10 (*p*-value = 0.0157), 12 (*p*-value = 0.0126), and 13 (*p*-value = 0.0048), suggesting an initial stage of rapid change followed by a stabilization phase. Previous studies have documented marked increases in phase separation after ten days of storage, mainly associated with coalescence processes [15], which is consistent with the observation of maximum separation around day 10 in this study. Subsequently, most formulations did not show substantial increases in the height of the separated phase, indicating that the system tended toward a new equilibrium. This separation has been linked to an increase in droplet size and greater interfacial tension between the oil and continuous phase [41].

**Figure 5:**
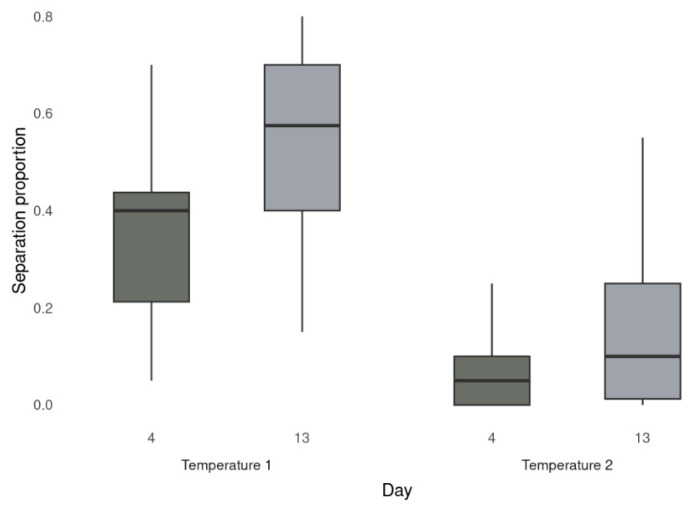
Separation proportion at the initial (Day 4) and final (Day 13) time points under two temperature conditions. Temperature 1 corresponds to ambient (room) temperature, and Temperature 2 corresponds to refrigerated conditions (4 °C).

Finally, heat treatment had a clear effect on stability, with significant differences (*p* < 0.00001) in phase separation between emulsions stored at room temperature and those stored under refrigeration. In all cases, separation was greater at higher temperatures, whereas samples stored at 4 °C showed much less destabilization, as presented in Figure 5. This behavior can be explained by the increase in the free energy of the system and the number of collisions between droplets at high temperatures, which favor coalescence and phase separation. At low temperatures, on the other hand, the speed and frequency of collisions decrease, reducing droplet size growth and slowing down destabilization processes [16,46].

### 2.5. Shelf-Life Evaluation

Paired tests were used, given the within-sample design on this study. No significant differences were detected before and after two months for emulsion stability metrics (ADS, PDI, and ζ-potential); small decreases were observed but were not significant (*p* > 0.05), consistent with good stability during storage at 4 °C. As noted above, refrigeration likely slowed the phase separation. In contrast, the inhibition diameter (ID) showed a significant decrease of approximately 37% after two months of storage (*p* < 0.05). This reduction indicates a relevant loss of antibacterial activity and should be interpreted with caution, particularly in relation to the potential shelf-life of the formulation, even though the emulsion retained significant anti-MRSA activity after storage (*p* = 0.00022). For comparison, in our stability monitoring of the non-encapsulated pigment extract, evaluated every seven days, the inhibition diameter decreased from 20.3 mm on day 1 to 12.0 mm after 40 days, corresponding to an approximately 41.0% reduction. This suggests that the loss of antibacterial activity may be partly associated with the intrinsic instability of the pigment, including sensitivity to light exposure [27], as well as other storage-related conditions. Similar losses have been reported for emulsified extracts; for example, a flower extract showed a decrease in inhibition zone from 27.76 mm to 17.34 mm within seven days [47], supporting the idea that extract-based systems may be particularly sensitive to storage conditions.

The observed decrease should therefore be considered a limitation of the current formulation for potential commercial application. These findings highlight the need to further evaluate the influence of storage conditions, including light exposure, temperature, oxygen availability, and packaging, on pigment stability and antibacterial performance. In addition, complementary antibacterial assays, such as MIC, MBC, time-kill kinetics, or release-based evaluations, would provide a more complete assessment of the bioactivity retained after storage since the disk diffusion method is influenced by compound solubility and diffusion through the agar medium.

Overall, although the emulsion retained significant anti-MRSA activity after storage, the approximately 37% reduction indicates that further formulation optimization, improved protection against pigment degradation, and long-term real-time and accelerated stability studies with predefined antibacterial potency acceptance criteria are required before its commercial suitability can be established.

### 2.6. Color Evaluation

A quadratic response surface model was fitted for total color difference (ΔE*), identifying pigment concentration and oil proportion as significant factors (adjusted R^2^ = 0.81), which indicates good interpretation. Because ΔE* integrates changes across L* (lightness), a* (red–green axis), and b* (yellow–blue axis), it is a practical indicator of perceptible color shifts; values of ΔE > 3* are generally visible to the human eye [36]. The same two factors (pigment concentration and oil proportion) were also significant for L*, a*, and h° (hue angle), whereas for b* and C* (chroma), a significant pigment-only concentration was found.

**Figure 6:**
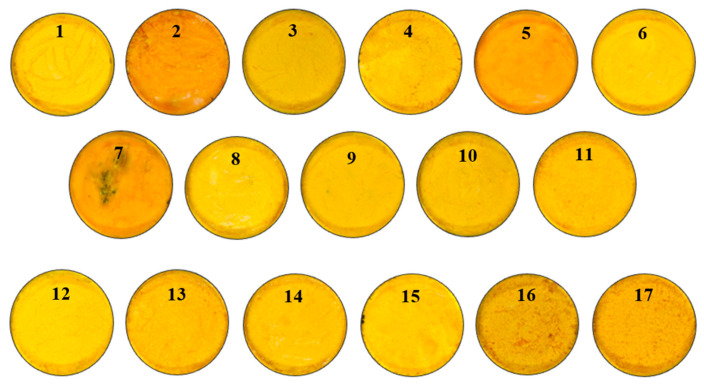
Representative images of the 17 emulsion formulations after two microfluidization cycles. Photographs were acquired in a dark box under standardized illumination using two fluorescent daylight lamps positioned at a 45° angle relative to the samples. Images were captured 17 cm from the samples with a Sony DSLR-A480 camera using Adobe RGB color space, ISO 1600, 35 mm focal length, and no flash.

Higher levels of the factors were associated with lower L* (lower lightness). This trend is consistent with reports that particle characteristics modulate light scattering and perceived lightness, where changes that reduce effective scattering decrease L* [48]. In parallel, a* increased (redder tones) and h° decreased (shift toward orange), an outcome that aligns with the intrinsic hue of the *Streptomyces* pigment extract and with the optical contribution of the HOPO oil, which tends to place hue angles near 80° [48]. For b*, higher pigment concentration yielded lower values, and C* also decreased, both coherent with the brownish character of the extract, which reduces yellowness and overall chroma, as shown in Figure 6. Finally, predictive equations were derived for each of the color variables described above.

### 2.7. In Vitro Cell Viability Assay by the MTT Method

Using the MTT methodology, it was possible to evaluate the viability of the three optimal emulsions for each case, the macroemulsion (ME) and the nanoemulsions with one and two cycles (C1 and C2), respectively, in both human dermal fibroblasts (HDFa) and human keratinocytes (HaCaT). Cell viability greater than 70% was found for the two cell types evaluated in the case of most emulsions, except for C1 in the HDFa cell line, indicating that the sample could be inducing cell damage, metabolic inhibition, or even cell death, which limits its potential use in applications with dermal contact, since values between 30% and 60% indicate moderate toxicity [49]. According to ISO/CD 10993-5 [50], values greater than 70% can be considered adequate for biomaterials as they would not be cytotoxic [49].

**Figure 7:**
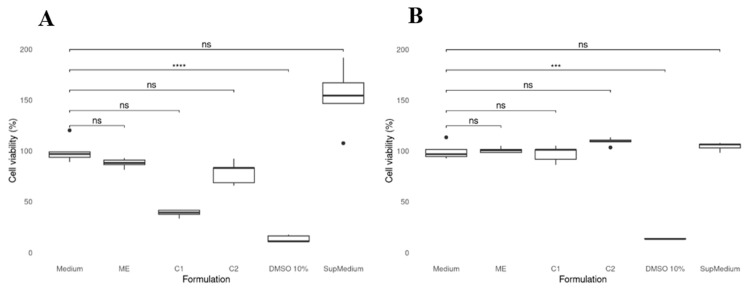
Cell viability of human dermal fibroblasts (HDFa) (**A**) and human keratinocytes (HaCaT) (**B**) after treatment with emulsion formulations (*n* = 5). Medium supplemented was used as a cell growth control (negative cytotoxicity control), while dimethyl sulfoxide (DMSO) at 10% served as positive cytotoxicity controls. ME: macroemulsion; C1: one cycle of microfluidization; C2: two cycles of microfluidization; SupMedium: supplemented medium; Medium: untreated medium. Significance levels: ns, not significant; *p* ≤ 0.001 (***); *p* ≤ 0.0001 (****).

HaCaT cells showed higher viability than HDFa cells, which is consistent with previous reports indicating that fibroblasts can be more sensitive than keratinocytes under similar exposure conditions, as shown in Figure 7 [51]. In addition, the pigmented extract has been reported to exhibit moderate cytotoxicity under environmental exposure, particularly light, since photoinduced reactions may promote structural rearrangements or oxidative degradation of the pigment [27]. Therefore, encapsulation may play an important role in protecting the pigment and reducing its direct interaction with cells, as suggested by the absence of cytotoxicity in most of the evaluated formulations.

Nevertheless, the cytotoxicity observed for C1 should be interpreted with caution. Although this response may be associated with greater pigment availability, lower colloidal stability, or differences in release behavior, specific release kinetics were not evaluated in the present study. Therefore, a direct causal relationship between zeta potential, pigment release rate, and cytotoxicity cannot be confirmed. In this context, zeta potential should be interpreted only as an indirect indicator of colloidal stability, since it may influence droplet aggregation, dispersion stability, and interactions with the surrounding medium [52]. The higher zeta potential and improved stability parameters observed for C2 may have contributed to a more stable dispersed system, whereas the lower stability of C1 could have favored greater exposure or availability of the pigment. However, this interpretation remains hypothetical and requires confirmation through encapsulation efficiency, release kinetics, and pigment degradation studies.

In the case of ME, although its stability parameters were less favorable than those of C2, its lower pigment loading and larger droplet size may have reduced the immediate availability of the active compounds, which could explain the lower cytotoxic response observed [52]. Overall, these findings suggest that formulation stability, pigment loading, droplet size, and surface charge may influence the biological response of the emulsions. However, further studies evaluating release profiles, pigment stability under storage and light exposure, and dose-dependent cytotoxicity are required to determine the mechanisms underlying the cytotoxic effect observed in C1.

## 3. Materials and Methods

### 3.1. Materials and Reagents

Yeast extract (Scharlab S.L., Sentmenat, Barcelona, Spain), Malt extract (Condalab, Madrid, Spain), Glucose (PanReac AppliChem, Barcelona, Spain), TSB (Condalab, Madrid, Spain), Agar-Agar (Velaquin, Mexico City, Mexico), HOPO was obtained from CENIPALMA (Bogotá, Colombia), food-grade non-purified soy lecithin (Manuchar Colombia Cía. S.A.S., Funza, Colombia), Vancomycin Antimicrobial Susceptibility discs (Oxoid Ltd., Basingstoke, UK), HDFa (Primary Dermal Fibroblast; Normal, Human, Adult ATCC^®^ PCS-201-012™, American Type Culture Collection [ATCC], Manassas, VA, USA), HaCaT (spontaneously immortalized human keratinocyte cell line derived from a distant periphery of malignant melanoma, ScienCell Research Laboratories, Inc., Carlsbad, CA, USA), MTT (3-(4,5-dimethylthiazol-2-yl)-2,5-diphenyltetrazolium bromide) (Invitrogen, Thermo Fisher Scientific Inc., Waltham, MA, USA), Trypsin-EDTA (0.25%) (Gibco™, Thermo Fisher Scientific Inc., Waltham, MA, USA), Fetal bovine serum (FBS) (Gibco™, Thermo Fisher Scientific Inc., Waltham, MA, USA), Dimethyl sulfoxide (DMSO) (Procell system ^®^, Procell Biotechnology Co., Houston, TX, USA), Methicillin-resistant *Staphylococcus aureus* (ATCC BAA44; American Type Culture Collection [ATCC], Manassas, VA, USA).

### 3.2. Bacterial Strain and Culture Conditions

*Streptomyces* sp. strain 145 (USABBIO collection, Universidad de La Sabana) was cultured on ISP2 agar composed of malt extract (10 g/L), yeast extract (4 g/L), glucose (4 g/L), and agar (20 g/L) [53]. Cryopreserved stocks (−80 °C; Revco, Thermo Scientific, Waltham, MA, USA) were thawed and reactivated in the same medium and, incubated for 7 days at 30 °C. Spores from each isolate were streaked on ISP2 agar and incubated for an additional 7 days at 30 °C FRIOCELL® incubator (MMM Medcenter Einrichtungen GmbH, Planegg, Germany). Three 1 cm^2^ plugs of fermented agar were transferred to 3 mL of liquid ISP2 medium and incubated for 7 days at 30 °C with orbital agitation at 150 rpm using a TE-4200 shaker incubator (Tecnal Equipamentos Científicos, Piracicaba, SP, Brazil). After this initial incubation, the culture was scaled up in several stages: first to 20 mL, then to 200 mL, and finally to 1000 mL. Each scaling step was conducted after a 7-day incubation period, using a 10% inoculum ratio at each stage. At each scaling step, antibacterial activity assays were performed to assess the inhibitory potential of the strain. Cultures were then centrifuged at 6000 rpm for 10 min at 4 °C to separate supernatants from biomass (HERMLE Labortechnik GmbH, Wehingen, Germany). Samples were frozen at −80 °C for 24 h (Thermo Scientific ultra-freezer) and subsequently lyophilized (Labconco Corporation, Kansas City, MO, USA) at 0.130 mbar and −53 °C for 3 days, including freezing, sublimation, and desorption phases. The resulting powder was reconstituted with 10 mL of sterile distilled water, mixed for 5 min with continuous stirring in an MX-S vortex mixer (DLAB Scientific Co., Ltd., Beijing, China), and centrifuged under the same conditions. The supernatant was lyophilized again for 3 days [27].

### 3.3. Pigment Characterization

#### 3.3.1. pH Titration

Two solutions, 0.25 M NaCl and 0.25 M HCl, were prepared for the titration process. A total of 1.5 g of extracted pigment was mixed with 15 mL of sterile water. The pH range from 2.18 to 11.9 was evaluated using these solutions and the Zetasizer Nano ZS (Malvern Instruments Ltd., Worcestershire, UK) with Zetasizer Nano software v3.30^®^. The program was first calibrated using the two solutions, after which the pH scan was initiated.

#### 3.3.2. Fourier-Transform Infrared Spectroscopy (FTIR-ATF)

Fourier Transform Infrared (FTIR) spectra were recorded using a Varian 630-IR spectrometer (Agilent Technologies, Santa Clara, CA, USA). Each sample underwent 16 scans, with a spectral range of 675 to 4000 cm^−1^ and a resolution of 4 cm^−1^. The analysis was performed using an Attenuated Total Reflectance (ATR) system, equipped with a Germanium (Ge) crystal and an incident angle of 45° (Cary 630 FTIR, Agilent Technologies, Inc., Santa Clara, CA, USA) [54].

#### 3.3.3. Minimum Inhibitory Concentration of the Extracted Pigment

The antibacterial activity of the pigment extracted from *Streptomyces* was evaluated against methicillin-resistant *Staphylococcus aureus* (MRSA) using a concentration-dependent inhibition assay. The lyophilized pigment extract was reconstituted in sterile distilled water to obtain the following concentrations: 0.001%, 0.005%, 0.009%, 0.020%, 0.050%, 0.082%, 0.100%, 0.250%, 0.500%, 1.000%, 2.500%, 5.000%, 10.000%, 15.000%, 33.000%, and 50.000%.

For each concentration, the antibacterial assay was performed in triplicate. MRSA was inoculated onto TSA medium under standardized conditions, and the pigment solutions were applied to sterile discs. Plates were incubated at 37 °C for 24 h, after which the inhibition halo diameters were measured in millimeters. The mean inhibition diameter and standard deviation were calculated for each concentration.

The minimum inhibitory concentration (MIC) was defined as the lowest pigment concentration that produced a measurable inhibition halo against MRSA under the evaluated experimental conditions. The concentration-dependent inhibition data were also used to estimate the IC_50_, defined as the pigment concentration required to reach 50% of the maximum inhibitory response. Data processing, descriptive statistical calculations, dose–response curve fitting, MIC and IC_50_ estimation, and graphical representation were performed using RStudio.

### 3.4. Emulsions Preparation

The aqueous phase, consisting of water and pigment extract, was dissolved using a vortex mixer for 2 min. Simultaneously, lecithin and HOPO were mixed at 7000 rpm using an Ultra-Turrax (IKA Works, Inc., Wilmington, NC, USA) for 1 min. The aqueous phase was then gradually added to the oil phase at the same speed for 3 min to form a macroemulsion. The emulsion was subsequently processed using a microfluidizer (LM10, Microfluidics, International Corporation, Westwood, MA, USA) for two cycles, according to a Box–Behnken experimental design using Design Expert software (version 10.0.1, Stat-Ease Inc., Minneapolis, MN, USA). Three factors were evaluated: pigment concentration (0–7%), oil proportion (80–90%), and microfluidization pressure (0–20,000 psi), as shown in Table 4.

The Box–Behnken design was selected because it allows for the evaluation of linear, quadratic, and interaction effects among three formulation and processing factors using a reduced number of experimental runs [55]. This approach was appropriate for studying the influence of pigment concentration, oil proportion, and microfluidization pressure on the physicochemical and antibacterial behavior of the emulsions, as well as for identifying the formulation conditions that provided the best overall performance. the formulation conditions that provided the best overall performance in terms of average droplet size, polydispersity index, zeta potential, physical stability, and antibacterial activity. The design was applied at three processing stages: macroemulsion after Ultra-Turrax mixing (ME), nanoemulsion after one microfluidization cycle (C1), and nanoemulsion after two microfluidization cycles (C2). This sequential approach was used to determine how the progressive energy input during microfluidization affected the emulsion properties and to verify whether additional processing cycles improved nanoemulsion formation and stability.

One of the five replicated center-point runs was excluded because it showed an unusually high residual during the diagnostic analysis compared with the remaining replicated runs, indicating a lack of consistency with the experimental variability observed for the center point. The observation was therefore considered a potential experimental deviation rather than a representative replicate. To avoid compromising data integrity, the exclusion was evaluated together with the residual diagnostics and the consistency of the remaining replicated runs. The final model was then fitted using the valid replicates, and the excluded value was reported as an outlier associated with experimental variability.

### 3.5. Emulsion Analysis

#### 3.5.1. Average Particle Size, Polydispersity Index, and Zeta Potential Analysis

Average particle size (ADS), polydispersity index (PDI), and zeta potential (ζ-potential) were measured for macroemulsion and nanoemulsion after the first and second cycles using a Zetasizer Nano ZS (Malvern Panalytical Ltd., Malvern, Worcestershire, UK) running Zetasizer Nano software v3.30^®^. The samples were diluted 1:1000 (*v*/*v*) prior to analysis.

#### 3.5.2. Antibacterial Activity

Antibacterial activity was evaluated using the disc diffusion method against methicillin-resistant *Staphylococcus aureus* (MRSA) [56]. Briefly, sterile paper discs were impregnated with 90 µL of the emulsion and placed on Tryptic Soy Agar (TSA) plates previously inoculated with 30 µL of the pathogen at 0.5 McFarland standard. The plates were incubated at 37 °C for 24 h, and the antibacterial activity was assessed by measuring inhibition zone diameters (Dh, mm) around the discs. Zones were categorized as: Dh < 10 mm, low activity (+); Dh = 10–20 mm, moderate activity (++); Dh > 20 mm, high activity (+++) [39]. Control discs were included in each assay: distilled water (negative control) and vancomycin discs containing 5 µg (positive control).

### 3.6. Physical Stability of Emulsions over Time at Different Temperatures

Stability was evaluated for the 17 emulsion formulations obtained in the second microfluidization cycle (C2), corresponding to the experimental runs established in the Box–Behnken response surface design. For each formulation, 10 mL aliquots were dispensed into falcons and stored under two conditions in duplicate (*n* = 2 per condition): ambient temperature and refrigerated (4 °C). The extent of phase separation was measured daily for two weeks to evaluate phase separation behavior at two different storage temperatures.

### 3.7. Shelf-Life Evaluation

After two months of refrigerated storage at 4 °C (RSA1UHSL1, Samsung Ltd., Suwon, Republic of Korea), all 17 emulsions corresponding to the second-cycle design (C2) established by the Box–Behnken response surface design were re-evaluated for average droplet size (ADS), polydispersity index (PDI), zeta potential (ζ-potential), using Zetasizer Nano ZS (Malvern Panalytical Ltd., Malvern, Worcestershire, UK), and inhibition diameter (ID). Pre- and post-storage values were compared using paired tests: when normality (Shapiro–Wilk) was satisfied, a paired t test was applied; otherwise, the Wilcoxon signed-rank test was used. Statistical significance was set at α = 0.05.

### 3.8. Color Analysis

Images were acquired with a Sony DSLR-A480 (Adobe RGB, ISO 1600, 35 mm focal length, no flash) positioned 17 cm from the samples, against a black background. All 17 emulsions corresponding to the second-cycle design (C2) established by the Box–Behnken response surface design were photographed under identical settings. Images were processed in ImageJ (NIH, e.g., v1.53) and converted to the CIELAB (CIE L*a*b*) color space (illuminant D65, 2° observer) using EasyRGB. From the L*, a*, b* values, chroma (C*), hue angle (h°) (reported in degrees), and color difference relative to the untreated control (CS) were computed using the following equations:

(1) $$C_{ab}^{*}=\sqrt{{\left(a^{*}\right)}^{2}+{\left(b^{*}\right)}^{2}}$$

(2) $$h_{ab}^{*}=\tan^{-1}\left(\frac{b^{*}}{a^{*}}\right)$$

(3) $$\Delta E=\sqrt{{\left({\Delta L}^{*}\right)}^{2}+{\left({\Delta a}^{*}\right)}^{2}+{\left({\Delta b}^{*}\right)}^{2}}$$

### 3.9. In Vitro Cell Viability Assay by the MTT Method

Human Dermal Fibroblast (HDFa) and human keratinocytes (HaCaT) cell lines were grown in DMEM supplemented with 10% fetal bovine serum in cell culture flasks and cultured at 37 °C and 5% CO_2_. Cell viability was assessed using the MTT method defined by ISO 10993-5 [50]. Cells at a concentration of 1.0 × 10^4^ cells per well were seeded in 96-well plates with supplemented medium at 37 °C and 5% CO_2_ for 24 h. Then, the medium was removed, and 100 µL of a 1% (*v*/*v*) solution of the optimized emulsion selected from each experimental design—macroemulsion (ME), one microfluidization cycle (C1), and two microfluidization cycles (C2)—was prepared in fresh medium and added to each well containing 100 µL of medium. The cells were left for 24 h at 37 °C and 5% CO_2_. After this, the supernatant and samples were removed, and the MTT solution (12 mM in PBS) was added in a total volume of 100 μL for 4 h at 37 °C. The supernatant was removed and 100 μL of dimethyl sulfoxide was added and incubated for 15 min at 37 °C. Absorbance was measured at 570 nm. Controls used were cells cultured with medium and cells with 10% dimethyl sulfoxide (DMSO) [49].

### 3.10. Statistical Analysis

Data were first assessed for normality (Shapiro–Wilk) and homoscedasticity (Levene’s test). If both assumptions were met, groups were compared by one-way ANOVA followed by Tukey’s HSD post hoc test. If assumptions were not met, the Kruskal–Wallis test was applied, followed by Dunn’s post hoc test (with appropriate multiple-comparison adjustment). Statistical significance was set at α = 0.05. All statistical analyses were conducted using RStudio version 4.5.1 (13 June 2025).

## 4. Conclusions

This study demonstrates that the pigment extracted from *Streptomyces* exhibits relevant antibacterial activity against methicillin-resistant *Staphylococcus aureus* (MRSA) and can be successfully incorporated into nanoemulsion systems using microfluidization. Pigment production was maintained during culture scale-up, with the highest antibacterial response observed between the third and fourth weeks of cultivation, particularly in the supernatant fraction. The extracted pigment showed a concentration-dependent antibacterial effect, with an MIC of 0.05% and an IC_50_ of 0.293%. In addition, pH-dependent analysis indicated greater pigment stability within the range of 5 to 8, which is compatible with potential topical and cosmeceutical applications. Importantly, the in vitro cytotoxicity assay showed that the pigment-loaded nanoemulsions were not cytotoxic to skin cells under the evaluated conditions, supporting their preliminary biocompatibility.

Nanoencapsulation by microfluidization proved to be an effective strategy to improve the physical stability of the pigment while preserving its biological activity. Increasing the microfluidization pressure reduced droplet size and polydispersity and improved zeta potential, with the best performance observed at 20,000 psi after two processing cycles. The nanoemulsions remained physically stable during two months of refrigerated storage, although antibacterial activity showed a slight decrease over time. Overall, these findings provide in vitro evidence that microfluidized nanoemulsions can act as protective delivery systems for *Streptomyces*-derived pigments, offering a promising platform for the development of multifunctional cosmeceutical formulations with antibacterial activity, physical stability, and preliminary skin-cell compatibility.

## Figures and Tables

**Table 1:** ANOVA summary and model fit statistics for the Box–Behnken design: Effects of independent factors on ID, ADS, PDI, and ζ-potential of the nanoemulsion after the second microfluidization cycle (C2). ID: inhibition diameter; ADS: average droplet size; ζ: zeta potential; PDI: polydispersity index. Bold values indicate statistically significant factors with *p*-values less than 0.05.

	ME								C1								C2							
Independent Factor	ID (mm)		ADS (nm)		PDI (nm)		ζ (mV)		ID (mm)		ADS (nm)		PDI (nm)		ζ (mV)		ID (mm)		ADS		PDI (nm)		ζ (mV)	
	SS	*p*-Value	SS	*p*-Value	SS	*p*-Value	SS	*p*-Value	SS	*p*-Value	SS	*p*-Value	SS	*p*-Value	SS	*p*-Value	SS	*p*-Value	SS	*p*-Value	SS	*p*-Value	SS	*p*-Value
Model	303.76	**7.82 × 10^−6^**	741,074.48	**3.69 × 10^−4^**	0.18	**4.38 × 10^−3^**	1178.47	**1.32 × 10^−2^**	295.56	**6.86 × 10^−4^**	382,159.80	**0.02**	0.11	**0.03**	1239.43	**3.49 × 10^−3^**	300.66	**0.01**	330,759.23	**0.02**	0.13	**0.01**	1823.06	**1.43 ×** **10^−3^**
A	259.81	**3.79 × 10^−7^**	435,209.51	**9.33 × 10^−5^**	0.13	**9.32 × 10^−4^**	297.68	**4.23 × 10^−2^**	249.52	**1.99 × 10^−5^**	10,954.47	0.27	2.26 × 10^−3^	0.44	105.37	**0.03**	272.42	**1.07 × 10^−4^**	3655.84	0.54	2.21 × 10^−4^	0.80	96.49	0.08
B	10.96	**0.04**	19,883.50	0.21	3.30 × 10^−3^	0.49	832.32	**2.75 × 10^−3^**	6.30	0.06	59,860.88	**0.03**	0.02	0.06	89.89	**0.04**	5.72	0.24	11,822.09	0.28	3.47 × 10^−3^	0.33	2.49	0.75
C									1.74	0.25	176,670.54	**2.74 × 10^−3^**	0.07	**3.04 × 10^−3^**	353.56	**1.92 × 10^−3^**	0.19	0.82	228,561.24	**9.52 × 10^−4^**	0.08	**9.68 × 10^−4^**	1150.80	**1.09 ×** **10^−4^**
AB	1.23	0.44	192,699.05	**1.92 × 10^−3^**	0.04	**0.03**	8.70	0.70	0.64	0.47	8942.85	0.31	1.40 × 10^−3^	0.54			0.25	0.80						
AC									1.10	0.35	68,251.56	**0.02**	0.01	0.09	247.01	**4.62 × 10^−3^**	0.23	0.80	46,160.52	0.05	0.02	**0.03**	184.96	**0.02**
BC									0.83	0.41	15,643.76	0.20	3.72 × 10^−3^	0.33	21.39	0.24	1.78	0.50	10,545.58	0.31	0.01	0.23	4.73	0.66
A^2^	27.78	**0.003**	46,129.20	**0.07**	0.01	0.26			34.83	**2.12 × 10^−3^**	5482.33	0.42			23.17	0.23	16.95	0.07	12,437.83	0.27	1.48 × 10^−3^	0.52		
B^2^	3.99	0.18	47,153.22	**0.07**			39.77	0.42	1.42	0.29					144.97	**0.02**	1.69	0.51					39.64	0.23
C^2^									0.57	0.49	38,207.52	0.06	0.01	0.24	225.64	**0.01**	1.44	0.54	17,576.13	0.20	0.01	0.13	343.95	**4.92 ×** **10^−3^**
Lack of Fit	4.41	0.58	38,354.44	0.37	0.03	0.37	260.63	0.37	2.04	0.75	27,605.95	0.61	0.02	0.34	41.22	0.71	7.22	0.68	54,350.77	0.30	0.02	0.04	33.14	0.97
R^2^	0.94		0.87		0.72		0.65		0.98		0.90		0.82		0.94		0.94		0.82		0.83		0.91	
R^2^-Ajusted	0.91		0.80		0.62		0.53		0.95		0.76		0.65		0.86		0.84		0.67		0.68		0.83	

**Table 2:** Predictive equations for ID, ADS, ζ-potential, and PDI of the macroemulsion and nanoemulsions after the first and second microfluidization cycles, with the current factors. ME: macroemulsion; C1: one-cycle emulsion; C2: two-cycle emulsion; ID: inhibition diameter; ADS: average droplet size; ζ: zeta potential; PDI: polydispersity index; L*: lightness; a*: red–green axis; b*: yellow–blue axis; h°: hue angle; C*: chroma; ΔE: total color difference.

Equations for …	Macroemulsion (ME)	1 Cycle (C1)	2 Cycles (C2)
ID (mm)	28.09 + 1.56A − 0.31B	15.49 + 1.57A − 0.16B − 5.42 × 10^−5^C	–188.01 + 3.98A + 4.43B + 9.75 × 10^−4^C − 0.014AB + 6.83 × 10^−6^AC − 1.33 × 10^−4^BC − 0.17A^2^ − 0.026B^2^ + 6.01 × 10^−9^C^2^
ADS (nm)	−3147.52 + 1348.84A + 41.39B − 15.10AB	−9.95 + 41.37A + 5.16B − 0.11C − 0.0033AC + 0.0013BC	765.97 + 4.94A − 2.58B − 0.11C − 3.07 × 10^−3^AC + 1.03 × 10^−3^BC + 4.55A^2^ + 6.63 × 10^−7^C^2^
ζ (mV)	125.41 + 1.66A − 2.01B	−1949.15 + 3.33A + 45.48B − 0.0014C − 2.28 × 10^−4^ AC − 0.27B^2^ + 7.97 × 10^−8^C^2^	−944.25 + 2.94A + 21.30B − 4.22 × 10^−3^C − 1.94 × 10^−4^AC + 2.17 × 10^−5^BC − 0.13B^2^ + 9.27 × 10^−8^C^2^
PDI (nm)	−2.13 + 0.63A + 0.03B − 0.007AB	−0.26 + 0.0095B − 6.34 × 10^−6^C	−0.797 + 9.01 × 10^−3^A − 3.15 × 10^−3^B − 7.45 × 10^−5^C − 2.15 × 10^−6^AC + 7.32 × 10^−7^BC + 1.57 × 10^−3^A^2^ + 4.81 × 10^−10^C^2^
L*	-	-	167.09 + 0.71A − 1.04B − 9.42 × 10^−4^C + 4.46 × 10^−5^AC + 1.17 × 10^−5^ BC − 0.32A^2^ − 1.08 × 10^−8^C^2^
a*	-	-	–99.59 − 0.93A + 1.19B + 3.21 × 10^−3^C + 4.70 × 10^−5^BC + 0.27A^2^ + 3.35 × 10^−8^C^2^
b*	-	-	332.31 + 7.66A − 6.06B + 1.19 × 10^−4^ C − 0.088AB − 0.16A^2^ − 0.036B^2^
C*	-	-	67.04 + 8.50A + 0.11B + 1.23 × 10^−4^C − 0.097AB − 0.15A^2^
h°	-	-	167.90 + 0.72A − 0.93B − 2.56 × 10^−3^C + 3.75 × 10^−5^BC − 0.21A^2^ − 2.72 × 10^−8^C^2^
ΔE	-	-	–103.65 − 0.36A + 1.32B + 2.59 × 10^−3^C − 4.04 × 10^−5^BC + 0.26A^2^ + 3.86 × 10^−8^C^2^

**Table 3:** Comparison of model predictions for three designs (ME, C1, and C2) across four response variables—ten in the case of C2—under optimized experimental conditions. ME: macroemulsion; C1: one-cycle emulsion; C2: two-cycle emulsion; ID: inhibition diameter (mm); ADS: average droplet size (nm); Zp: zeta potential (mV); PDI: polydispersity index (nm); L*: lightness; a*: red–green axis; b*: yellow–blue axis; h°: hue angle; C*: chroma; ΔE: total color difference.

Emulsion	Factors			Desirability		Values		Error (%)
	A	B	C			Model	Experimental	
ME	3.9	89.5	-	0.71	Zp	−48.1	−50.7	5.2
					PDI	0.566	0.577	1.9
					ADS	531.3	586.8	10.5
					ID	7.5	9.0	20.0
C1	6.0	80.0	16,520	0.94	Zp	−51.0	−52.8	3.5
					PDI	0.355	0.357	0.7
					ADS	223.1	230.1	3.1
					ID	11.7	11.3	3.1
C2	7.0	80.1	19,711	0.95	Zp	−65.1	−58.9	9.5
					PDI	0.262	0.250	4.5
					ADS	168.6	186.2	10.4
					ID	13.4	13.0	3.0
					L*	75.23	69.53	7.6
					a*	4.79	5.20	8.5
					b*	75.85	68.69	9.4
					C*	86.03	85.67	0.4
					h°	75.93	68.99	9.1
					ΔE	14.95	14.00	6.3

**Table 4:** Box–Behnken response surface design for macroemulsion, one-cycle emulsion, and two-cycle emulsion systems with three experimental factors and multiple response variables across 17 experimental runs. ME: macroemulsion; C1: one-cycle emulsion; C2: two-cycle emulsion; ID: inhibition diameter; ADS: average droplet size; ζ: zeta potential; PDI: polydispersity index; L*: lightness; a*: red–green axis; b*: yellow–blue axis; h°: hue angle; C*: chroma; ΔE: total color difference. A: Pigment concentration (% *w*/*w*), B: Oil proportion (% *w*/*w*), C: Microfluidization pressure (psi).

			ME				C1				C2									
A	B	C	ID (mm)	ADS (nm)	ζ (mV)	PDI (nm)	ID (mm)	ADS (nm)	ζ (mV)	PDI (nm)	ID (mm)	ADS (nm)	ζ (mV)	PDI (nm)	L*	a*	b*	h°	C*	ΔE
3.5	80	0	9.19	607.00	−36.50	0.57	9.48	607.00	−36.50	0.55	9.19	607.00	−36.50	0.55	84.35	−5.18	75.98	93.91	76.16	3.78
7.0	85	0	12.75	922.43	−22.10	0.75	12.44	902.35	−21.37	0.75	12.44	902.35	−20.85	0.75	67.80	11.55	71.40	80.80	72.36	21.70
3.5	85	10,000	10.62	387.30	−41.15	0.55	9.75	1316.00	−37.03	0.86	9.82	431.85	−58.45	0.44	75.05	−1.90	73.30	91.48	73.33	7.26
7.0	90	10,000	9.55	580.70	−50.05	0.57	10.15	515.05	−50.20	0.49	9.93	492.07	−55.55	0.39	66.90	1.47	67.96	88.77	68.10	16.60
7.0	85	20,000	11.53	757.95	−30.80	0.71	10.35	269.00	−47.55	0.39	13.40	243.35	−59.45	0.33	72.10	11.62	71.70	80.81	72.63	19.29
3.5	85	10,000	8.75	438.45	−32.68	0.51	10.57	299.30	−46.43	0.42	9.62	306.55	−62.10	0.46	83.53	−4.45	74.71	93.44	74.85	2.43
3.5	90	0	7.64	549.57	−42.60	0.53	7.64	574.05	−41.85	0.56	7.64	549.57	−36.75	0.53	71.41	8.25	72.37	83.50	72.89	16.81
3.5	80	20,000	9.80	561.30	−15.70	0.40	9.57	259.60	−48.00	0.37	9.44	272.35	−61.65	0.34	81.88	−4.49	79.71	93.25	79.85	6.33
0.0	85	0	0.00	465.95	−45.05	0.48	0.00	531.15	−45.10	0.53	0.00	531.15	−45.10	0.53	77.92	−0.75	76.96	90.55	76.97	6.70
0.0	85	20,000	0.00	256.85	−40.15	0.40	0.00	420.30	−39.85	0.40	0.00	301.85	−56.50	0.41	75.97	−1.70	75.81	91.25	75.86	6.99
3.5	85	10,000	9.66	606.00	−44.05	0.55	8.73	397.60	−53.67	0.48	5.91	441.55	−47.65	0.41	78.81	0.96	80.08	89.31	80.10	9.42
0.0	80	10,000	0.00	361.60	−37.00	0.43	0.00	223.10	−53.27	0.41	0.00	454.40	−59.10	0.46	81.58	−5.10	78.71	94.03	78.41	0.00
3.5	85	10,000	10.35	693.25	−45.53	0.66	7.39	763.50	−52.17	0.74	5.83	249.75	−54.60	0.42	77.77	−0.86	79.88	90.65	79.05	6.75
3.5	90	20,000	4.20	690.05	−48.60	0.74	5.91	476.80	−62.60	0.50	5.21	420.30	−57.55	0.47	71.29	−0.46	79.04	90.35	79.89	11.29
0.0	90	10,000	0.00	565.50	−61.25	0.56	0.00	571.55	−58.33	0.56	0.00	520.00	−59.93	0.52	75.24	3.29	78.38	87.46	78.81	10.61
7.0	80	10,000	11.77	1254.75	−31.70	0.84	11.74	355.73	−48.40	0.41	10.92	340.65	−57.00	0.40	73.91	−1.56	74.43	91.18	74.49	8.50
3.5	85	10,000	6.83	466.95	−42.20	0.50	8.19	482.13	−46.27	0.50	6.32	310.55	−48.75	0.44	76.34	−0.64	75.45	90.49	75.50	7.18

## Data Availability

The raw data supporting the conclusions of this article will be made available by the authors on request.
